# Motorcycle Taxis, Extended Lockdown and Inequality at Work in Kampala, Uganda

**DOI:** 10.1007/s12132-022-09468-6

**Published:** 2022-07-22

**Authors:** Richard Mallett, Lillian Asingura, Geofrey Ndhogezi, Disan Byarugaba, Hakimu Sseviiri

**Affiliations:** 1 Department of International Development, London School of Economics, London, UK; 2 Department of Geomatics and Land Management, Makerere University, Kampala, Uganda; 3 Motorcycle Taxi Rider and Independent Researcher, Kampala, Uganda; 4 Department of Geography, Geo-Informatics and Climatic Sciences, Makerere University, Kampala, Uganda; 5 Sustainable Development Solutions Network Uganda, Kampala, Uganda

**Keywords:** Motorcycle taxis, Livelihoods, Labour, Informality, Platform work, COVID

## Abstract

After two years of extended lockdown, Kampala’s vast workforce of motorcycle taxi riders today looks a little different. Though the sector has long constituted a vital source of labour and income for many thousands of urban residents cut off from more decent opportunities elsewhere in the economy, a recent combination of lockdown pressures and digital transitions has created new forms of dependency upon the sector whilst simultaneously stripping some old ones away. In this article, we draw on in-depth qualitative data from interviews with riders, carried out at different stages of the pandemic, to show how the composition of labour within the sector has been reworked by a series of ‘selective exits’ and ‘substitution effects’ over the past two years. In exploring the nature and nuances of these parallel movements, our analysis not only reveals considerable socio-economic unevenness within the city’s motorcycle taxi sector itself but also sheds light on a new, broader configuration of urban inequality in the making.

## Introduction

For close to two years during the COVID-19 pandemic, Uganda’s motorcycle taxi sector took a battering. What started out as a 14-day government-imposed suspension of public transport and passenger ferrying towards the end of March 2020, implemented alongside a wider raft of temporary measures designed to curb the virus’ spread in those early days of the pandemic, ended up as a shifting regime of lockdown and curfew that lasted 22 months. It was not until 7 February 2022, one month after schools reopened and two weeks after Uganda’s vibrant nighttime economy was resuscitated by the lifting of a 7pm curfew on the general population, that government restrictions on motorcycle taxis were finally dropped. This ubiquitous form of popular transport, known locally and regionally as boda bodas, was the last piece of the economy to be fully restored.

Uganda’s boda boda sector is one of the country’s most significant labour markets. Though accurate statistics do not exist, it is generally understood that boda riding constitutes the single largest sector of male employment outside agriculture (Amone, 2021).^1^ Relatively recent estimates suggest there are 145,000 boda riders in Kampala alone (Evans et al., 2018), though the number could be as high as 300,000 (Doherty, 2020) or even 400,000 (Saturday Vision, 2022), and that the industry provides income to about 25% of the capital’s population in one form or another (Evans et al., 2018).^2^

For all the stigma attached to boda riding, particularly so in the congested and youthful contexts of large towns and cities, this is a sector of ‘vital’ economic activity in more ways than one (Fredericks, 2014). As a means of ‘unparalleled agility in heavy traffic’ and an integral part of an underfunded public transport system (Goodfellow & Mukwaya, 2021: vi), the informal work performed by boda riders enables much-needed connectivity and convenience for a wide cross-section of residents, commuters and businesses, and in so doing constitutes ‘essential labor at the base of urban development’ (Fredericks, 2014: 534). There is a vitality too in the way that the transport infrastructure contributed by motorcycle taxis is literally embodied in the lives of boda riders (Doherty, 2017), who on a daily basis expose themselves to levels of physical risk that are so high the largest public hospital in the country reportedly has a dedicated boda crash department (Evans et al., 2018: 676).^3^ And at the level of livelihoods, this is a sector that absorbs huge numbers of people cut off from the limited number of better paid opportunities for decent work elsewhere in the economy, providing an indispensable lifeline for those positioned at the hard edge of jobless growth (Khisa, 2019; Kjaer & Katusiimeh, 2012).

What have the last two years done to this vital yet locked-down sector of work and economic activity? How have the livelihoods of boda riders been affected, and what kinds of changes have been brought about by the government’s evolving set of rules and restrictions, from the suspension of passenger ferrying (which in the end persisted for four months rather than two weeks) to an enduring ban on riding after dark?

While Ugandan news outlets regularly featured coverage of these latest developments alongside a scattering of more analytically focused commentaries (e.g. Courtright, 2022; Muhindo, 2022; Onyango-Obbo, 2022),^4^ to date there has been little in-depth examination of what has been going on within the capital city’s boda boda sector, particularly that which is grounded in the concrete experiences of boda riders themselves and connected to broader questions of urban development and inequality. This article is one effort to help fill that largely empty space within the academic literature, drawing primarily on semi-structured interviews with riders working in Kampala under extended yet shifting lockdown conditions to shed light on wider transformations playing out across sector and city during this period.

The findings paint a picture of considerable unevenness in the way that COVID restrictions have affected work and livelihoods across Kampala’s urban economy, with our data suggesting a partial reconfiguration of the socio-economic composition of the city’s boda boda workforce—at least temporarily. This has been driven by a kind of parallel movement, which speaks in turn to a broader point about how inequality works *within* the sector itself. On the one hand, large numbers of individuals exited the industry as COVID restrictions extended over time, reflecting particular vulnerabilities attached to the nature of their working arrangement and economic position within the sector. On the other hand, individuals with little or no previous experience of boda riding entered the sector as a response to job losses and suspended salaries in the wider economy, in a sense substituting those who had little choice but to depart and replenishing the city’s supply of boda labour (albeit at a time when many of the ‘usual’ sources of demand for it were being crushed). Significantly, however, this does not appear to have been a like-for-like substitution. Many of those now turning to the motorcycle as a means of survival are arriving from elevated starting points relative to some of the industry’s most precarious and dependent longer-term riders, facilitated into the sector by an expanding array of possibilities for digital platform work and frequently bypassing pre-existing institutions of entrance and operation. Though the specific scale of this practice is difficult to determine, and while we must be careful about lumping all ‘exits’ and ‘entrants’ into discrete, cleanly bounded categories, there are both indications here of important social shifts within the city’s boda workforce itself alongside implications for how infrastructures of urban consumption and informal labour are being reworked and brought together in new ways (Meagher, 2021).

With regards to how the COVID pandemic has played out within the specific context of Kampala’s boda boda economy, the story emerging from our research is thus one of layered and nuanced effects. Rather than resulting in decline and dispossession across the board, what we instead find is a more complicated picture in which the government’s restrictions interacted with existing forms of unevenly distributed precarity to strip some (but not all) of their core livelihoods, whilst simultaneously manufacturing new dynamics and patterns of urban inequality. This story is developed over the course of the four following sections, starting with a brief overview of data and methods. We then, in separate sections, explore the two parallel processes outlined above—‘selective exits’ and ‘substitution effects’—in greater depth, drawing directly on the empirical data to illustrate how these changes have been unfolding and experienced by workers in Kampala’s boda economy. Finally, we conclude by drawing out some theoretical implications from the discussion.

## Methods and Data

This article is based on qualitative data generated through in-depth semi-structured interviews, carried out over an extended period whilst Uganda’s lockdown measures were still in place. They form part of the lead author’s PhD project, which looks at livelihoods, politics and the changing nature of work within Kampala’s boda boda sector. Although additional methods have also been used as part of this study, for our purposes here we draw specifically on interview material collected at two different points in time.

A first round of interviews was conducted by a small team of researchers at Makerere University’s Urban Action Lab towards the end of 2020, at a time when COVID restrictions prevented international travel and the completion of in-person fieldwork by the lead author. These initial interviews were organised through detailed online discussions between team members concerning the objectives of the exercise, the design of interview guides, sampling strategy, and plans for transcription and secure data storage. A total of 26 interviews with boda riders were carried out in November 2020, with researchers being careful to ensure variation across the sample of interviewees in order to capture diverse experiences and perspectives. To this end, rider selection was carried out with two key variables in mind: means of motorcycle access (i.e. those who own vs. those who either rent on a daily basis or are in the process of paying off a hire-purchase loan); and means of accessing jobs (i.e. those using a digital platform to find passengers and deliveries vs. those working completely ‘offline’). This basic framework generated four categories of boda rider, which are fairly evenly represented within the final sample (Table 1).

**Table 1:** Categorical breakdown of November 2020 interviewees

	Digital	‘Analogue’
Owner	5	8
Renter	6	7

Once these categories had been established, we made further efforts to reduce sampling bias by designing a selection strategy that to some extent reflected the urban geography of Kampala’s boda boda sector. Rather than focus on a single location, potential interviewees were approached at a range of sites within the city, including: (i) busy commercial areas of high economic activity (e.g. around malls, arcades, markets, high-traffic roads); (ii) popular hangout spots where riders would often congregate to eat, rest or chat amongst themselves, including at times when they were not actively working; and (iii) quieter areas with higher levels of residential land use. Though these sites were chosen purposively, the strategic selection of multiple areas in this way helped to construct a more realistic picture of the sector’s internal composition than would have been possible using either a single-sited approach or one based on selection by administrative unit.^5^ This principle was carried through the entirety of the project, informing the design of all subsequent research methods.

A second round of interviews took place between October 2021 and February 2022, once the easing of COVID restrictions permitted international travel. Though an interval of this length was unplanned, it made preliminary analysis of the earlier interview material possible and proved useful in structuring the next steps. In particular, it allowed us to: develop a clearer sense of what changes in the city and sector seemed to be unfolding amid lockdown conditions; think through appropriate ways of probing these issues further; and more generally ground the design of subsequent interviews in analysis of the earlier material. During this five-month period, 51 additional interviews were carried out with boda riders. These typically lasted between one and two hours, drawing on life-work history approaches to open up a dialogue about people’s journeys into the sector as well as exploring their current experiences and perceptions of life in the industry (Lewis, 2008). By contrast, our earlier round of interviews in November 2020 focused more on personal assessments of how things had changed within the sector since the start of the pandemic, what riders felt about this, and how they had been faring.

In the two sections that follow, we draw first on data from the initial set of November 2020 interviews to outline the beginnings of our story (‘selective exits’), before zeroing in on some selected accounts from our later interviews to develop a more detailed picture (‘substitution effects’). In particular, we use the in-depth recent experiences of one new entrant to the boda sector as a way of drawing out broader insights into the nuanced nature of change within the workforce during this time.

## Selective Exits

When our initial interviews were carried out in November 2020, the Ugandan government’s restrictions on boda activity had been in place for just over seven months. During this time, their severity shifted in response to changing assessments of the public health threat posed by the virus, starting out as an outright ban throughout the earliest phases of the pandemic before easing to permit the carrying of cargo but not passengers for several months. By the end of July 2020, riders were once again allowed to take passengers but with both a series of ‘standard operating procedures’ and a 6pm curfew in place. These partial lockdown conditions remained relatively stable until Uganda’s second wave hit in the second quarter of 2021, which resulted in a return to total lockdown from June onwards and a ban on passenger ferrying. By August 2021, infection rates had slowed and restrictions once again began to ease. For the rest of the year, riders could take both passengers and cargo until nightfall, and this was largely how the situation remained until all measures were lifted on 7 February 2022—a fortnight after the rest of the economy had been fully reopened.

Prior to the pandemic, the boda industry provided work and income to a colossal number of people, likely forming the country’s largest sector of urban employment for men. For at least the last two decades, it has kept numerous individuals afloat as an increasingly all-consuming politics of regime survival worked against sustained commitments to structural transformation and broad-based job creation (Golooba-Mutebi, 2020; Hickey et al., 2021). But that is not to say the work is desirable. Though it does nothing to undermine the economic value of the activity, there is a clear sense from many riders that their livelihoods are only motorcycle-based because there is little else available to them. One interviewee put it like this: ‘We are doing it because of poverty…Since I don’t have what to do, even in the following years I will be doing the same job’.^6^ What drives so many people’s participation in the boda economy is the absence of half-decent alternatives elsewhere in the informal economy that offer equivalent levels of pay, combined with widespread acceptance that office jobs and other ‘clean’ forms of employment within the formal economy are simply beyond reach without the right connections or backgrounds.

This might give the impression of a relatively homogenous workforce, but that could not be further from the truth. Although commonly portrayed as a job for the youth, boda work sucks in people of all ages. Educational backgrounds are similarly diverse, with the presence of secondary school completers and even university graduates calling into question a popular ‘conventional wisdom’ that boda riding is only for the country’s uneducated and illiterate. For example, from a survey of 370 riders carried out as part of this study, 9% had either fully or partially completed some form of further or higher education, whilst 6% had finished all levels of secondary school. By contrast, just 1% had received no formal schooling at all. The fact that Ugandans already in full-time (or close to full-time) employment have often strategically incorporated bodas into their working lives further bears out this picture of internal heterogeneity, with teachers in particular having long used intermittent riding to supplement their wages or get through periods of delayed pay.

An important distinction also exists in how working arrangements are differentiated among riders. Broadly speaking, there are three main routes to accessing a motorcycle for work. The first involves an outright purchase, either by putting accumulated savings to use or selling household assets to raise capital. One often hears stories about how people, especially young men, routinely sell off family or ancestral land in the village before heading to town to take up a ‘boda boda lifestyle’ (Branch, 2013: 3163). Indeed, this is another ‘conventional wisdom’ surrounding the industry, linked in turn to a powerful social narrative that characterises the rapid expansion of boda work as somehow symptomatic of deeper moral and cultural erosions within Ugandan society. A second option is to rent someone else’s motorcycle for a relatively fixed daily or weekly amount. Known locally in Kampala as the *kibaluwa* system, riders usually pay the owner 10,000 UGX a day (just under 3 USD) in exchange for access, and are responsible for purchasing their own fuel as well as handling minor maintenance work. While it is often claimed that the owners or ‘bosses’ embedded in this system tend to be wealthy, politically connected (or involved) individuals, again the reality is much more diverse. Purchasing one or two motorcycles and renting them out through the *kibaluwa* system is a widespread money-making strategy among members of the middle class looking for easy investment opportunities (Kayondo, 2020, 2021), and it is also common for boda riders themselves to hire out old motorcycles to colleagues. This ‘pay-as-you-go’ arrangement, which typically takes place on an informal basis and in most cases does not directly lead to riders becoming owners, has long been considered the dominant way through which individuals enter and operate within the sector. In contrast, the third main route involves entering into a hire-purchase or lease-to-own agreement, resulting—if all goes well—to eventual ownership of the motorcycle. These agreements are sometimes extensions or evolutions of the aforementioned rental arrangements, negotiated directly between parties within the *kibaluwa* system, but they also exist as a core financial product offered by a growing number of private companies and for-profit social enterprises. In recent years, asset financing has become central to how Uganda’s boda boda industry works, and the continued influx of foreign capital into this ‘frontier market’ suggests we are likely to see increasing levels of financialisation in years to come. There are already indications that hire-purchase arrangements, which essentially formalise the means through which riders acquire motorcycles whilst simultaneously generating new revenues for a wider set of external actors, are becoming the dominant route of access in Kampala and potentially further afield (Spooner et al., 2020). It is largely as a result of this that growing numbers of people are today able to move away from the old rental system and become ‘owner-riders’—or, to use the phrasing often heard during interviews (as well as in the promotional materials of loan companies), to ‘become their own boss’.

Soon after Uganda’s COVID restrictions were first introduced by President Museveni in March 2020, and later tightened in subsequent announcements, reports started coming through of thousands of city dwellers fleeing to the ‘safety of ancestral countryside homes’ in search of open space, cheaper living and more reliable food supplies (Onyango-Obbo, 2020; Waita, 2020). Mirroring scenes playing out across the world, this was not about an ‘emptying out’ of cities in an absolute sense, but rather a highly selective process in which the precariously positioned urban working poor felt little choice but to seek out whatever alternative systems of support they could. This was about survival, when the threads that hold together uncertain livelihoods are barely there anymore (Zeiderman et al., 2015).

Kampala’s boda industry acted as a kind of microcosm for what was going on in the wider city at the time, with selective urban–rural migrations drawing people away from, and in a sense decongesting, the workforce. When we asked people in November 2020 about what big changes they had seen in the sector since the pandemic began, this was one of the central themes to emerge. Interviewees routinely talked about how COVID restrictions had forced many people to leave the industry in order to find work and support elsewhere, both within but more frequently outside the city. They often drew links between a reduction in business on the one hand—as well as directly limiting the movements of riders, restrictions also sucked out the lifeblood of the boda economy in the form of passengers needing to go places, meaning for many months there were simply fewer jobs to go round—and dropouts from the sector on the other. This combination of shrinking demand and limited working hours produced one of the pandemic’s most pronounced impacts on the industry: a dramatic reduction in the daily incomes of riders. Estimates based on survey data collected in October 2020 suggest that during this period, riders’ average incomes had fallen by 35% compared to pre-COVID levels (Park et al., 2021). Had that research been carried out a few months earlier while full lockdown restrictions were still in place, the gap would have been even wider—and that more drastic situation would have characterised most riders’ experience of the first phase of the pandemic.

In an industry that demands substantial recurring costs to stay operational, this reduction in incomes had a major effect on the financial capacity of many riders to maintain their position in the economy. Some were able to weather the difficult circumstances by picking up new ways to generate income, or by moving pre-existing side hustles more centre stage. One interviewee shared how he had turned to brickmaking once the boda ban came in, explaining that ‘during the lockdown we were only working for food’.^7^ Another rider, a 22-year-old living on the outskirts of greater Kampala, told us how he was ‘gambling with fellow youths and playing cards to buy food for the family’, an activity that might have previously served to kill time or supplement income, but upon which this young man had come to depend during this difficult period.^8^

In cases like these, riders found themselves temporarily cut off from their primary source of work and income but were nonetheless able to stay afloat through other means before returning to their motorcycles when measures permitted them to do so. In other cases, however, it was simply not possible for riders to make that temporary switch whilst leaving their bikes ‘parked at home’ for five months, as one interviewee put it,^9^ reflecting internal differences within the industry concerning rental and ownership patterns. When we probed the issue of exits from the sector, interviewees pointed towards riders lacking reliable access to a motorcycle. Referencing that link between lower demand on the streets and increased financial pressures forcing selective exits, one owner-rider told us:

The change is much, since children no longer attend schools and few people are now going to churches...It has impacted on the number of passengers we now have, since a good number of people are not moving…Many riders have lost their boda boda to bosses since they couldn’t afford paying. This has resulted in the decrease.^10^

Another rider made similar remarks, suggesting that the worst effects of the pandemic were experienced by ‘colleagues [who] lost their boda bodas to the bosses and companies’.^11^ Comments like these illustrate an unevenly distributed precarity among Kampala’s boda workforce, which places some at a considerably higher risk of dispossession than others when the wider economy contracts. Though the prospect of becoming one’s own boss through motorcycle ownership is increasingly positioned as both viable and aspirational—a liberatory pathway to entrepreneurial empowerment in contexts where individuals are framed as job creators rather than job seekers (Dolan & Rajak, 2016)—getting there can be an uncertain journey. Asset finance companies enforce strict rules around loan repayments, including late-pay penalties on top of already-high interest rates, and when repossessions do occur their ‘clients’ tend to lose anything they have paid up until that point (which can be well in excess of 1,000 USD). At the height of the pandemic, some companies implemented grace periods or relaxed repayment schedules as a way of preventing defaults, but this was not universal and riders often brought up instances of unforgiving or inflexible practices—either as personal experiences or as things they had heard happen to others. For those using the *kibaluwa* system or engaged in hire-purchase arrangements with individuals rather than companies, dispossession during this period was a similarly real threat. While some interviewees would talk about how arrangements with individual owners offered more space for understanding and negotiation, ultimately the fates of riders in this segment of the labour market were and are at the discretion of the owners of the asset. In some situations, owners would repurpose their motorcycle(s) only as a last resort once other safety nets or financial avenues had been exhausted, selling it off to generate survival revenue or even becoming riders themselves. As one of our interviewees put it, it was ‘mostly those riding for [other] individuals [who] failed and left. Many bosses sold their bodas to sustain their families’.^12^ But in the worst cases, repossessions would reveal the capriciousness which Ettlinger (2021) argues often characterises power relations within informal workplaces, whereby renters are confronted with a ‘toxic mix of unpredictable actions at a whim and the absence of ethical considerations’.

These reports of dropouts from the sector are reflected in observations from other sources, including from within the digital ride-hailing space. In a media interview just three months into the pandemic, one of the founders of Uganda’s largest ride-hailing company, SafeBoda, said that 30% of the platform’s registered riders had left Kampala and returned to the village (Daily Monitor, 2020). And it does not seem like things improved in the year-and-a-half that followed, with key informant interviews with SafeBoda staff suggesting that over time as much as 80% of riders registered prior to the pandemic may have dropped off the platform.^13^

## Substitution Effects

In theory, the departure of working riders from Kampala should have created economic benefits for those who stayed, easing competitive pressures and in a sense helping the labour market recalibrate itself in light of COVID-induced compressions in demand. But that is not what appears to have happened. Instead of emptying out the sector, extended lockdowns produced a sizeable section of the urban population in need of back-up employment—and many of those affected turned to the motorcycle.

In interviewees’ accounts of recent change within the industry, their statements about high levels of dropout were often qualified with the observation that many newcomers seem to have arrived during the same period, somehow undoing the theoretical silver lining created by the departure of former colleagues and counterparts. In the words of one rider, ‘Some left [since the start of the pandemic] but on percentage the number of those joined is high, since other professionals…are on a stand-still and resorted to riding boda bodas’.^14^ Interviewees’ comments pointed to a kind of ‘substitution effect’ that had been taking place within the urban workforce, and that was potentially still ongoing, in which those who had either voluntarily or involuntarily exited Kampala’s boda sector were essentially being replaced by a fresh wave of new riders. When interviewees talked about these new arrivals, they often suggested that people in this category were tending to come from sectors or backgrounds not typically associated with an entrance into boda work, as the following quote illustrates: ‘We are still in the lockdown. Even the educated think when they get into riding boda boda, at least they can get food for their houses. I know one [studying] at Uganda Christian University in Mukono, but he is [now] in boda boda’.^15^ Many commented specifically on the entrance of teachers into the industry, with the closure of Uganda’s schools for close to two years displacing thousands into the wider labour market (Mwesigwa, 2021), while others mentioned security guards and professional drivers who had been laid off by employers and factory workers whose plants had shut.

Though an interesting theme to emerge from our November 2020 interviews, it had not been possible at the time to dig more into the lives of the sector’s new entrants. Who were these people, beyond their previous occupational identities? What were their specific starting points, and what routes did they take to get into the industry? How had they been finding it so far?

When the main phase of fieldwork for this project was finally permitted in the second half of 2021, we had the opportunity to go back and ask these questions—not just to those with long histories in the sector, but to people at the very centre of this dynamic. Throughout the course of interviewing between October 2021 and February 2022, we were able to probe the theme of COVID-related exits and entrances from a slightly more longitudinal perspective, exploring the extent to which these appeared to be sustained changes and what, if any, further consequences they had been having. We also met a number of riders in the new arrival ‘category’, and used those conversations to shed light on aspects of their work histories, experiences of urban life under lockdown, and plans for the future.

Samuel was one such rider.^16^ Originally from Buyende in eastern Uganda, he first came to Kampala several years ago to further his teaching career. In the decade or so that he has been in the capital, he has shifted jobs a lot, doing stints at different schools whilst always on the lookout for new personal connections that would help him secure more lucrative positions at better establishments. An ambitious character, he looks back at himself during those years ‘as someone who was moving, to a certain extent’. By the time the pandemic hit, he was earning a monthly salary of 900,000 UGX (approximately 250 USD), with aspirations to soon become a school director.

Shortly after the school closures, he and a few fellow teachers decided that they were ‘going to break the law’ by knocking on doors and offering private tuition services to any household who might be able to afford them. This proved only to be a stop-gap measure, however, helping out for the first few months but drying up soon after. As lockdown endured and the Presidential announcements kept coming, Samuel took stock of his responsibilities as a husband and father, assessed the options before him, and realised he ‘needed to shift’. That was when the boda came in.

Samuel already owned a motorcycle, which he had been using as a form of personal transport for a few years. He had never previously pictured himself riding to earn income. Neither had his wife who initially pushed back against his suggestion to enter the sector, asking him how he could even think about ‘leaving a smart job and going to do a dirty job’ and pointing out how dangerous the work could be—something he was also aware of. A year-and-a-half later, their perspectives are a little different. ‘I can see that there is money in boda boda’, he explained, telling us how ‘with the clean job I can get 900k [a month], but with the dirty job I can get 1.2 million. Even my wife has already changed her mind about it!’

Samuel’s reported earnings sit right at the upper end of what a boda rider in Kampala can expect to earn, even after several years of accumulating experience and contacts, and his testimony provides a few clues as to why he has been able to reach this status in such a short period of time. First, because of his relatively privileged starting point, Samuel managed to bypass both the *kibaluwa* rental system as well as the long road to ownership via a hire-purchase arrangement, thus substantially reducing his operating costs and maximising his take-home pay from day one. Though we came across a few others in this position during the course of our interviews, it seemed relatively uncommon for new riders to already be in possession of a fully paid-up motorcycle before starting out. In addition to those choosing to feel out the work by renting on a daily basis and seeing what sort of money they could turn over, generally speaking new riders would instead be those capable of either making the sizeable upfront payment required to enter a formal hire-purchase arrangement, or buying a second-hand motorcycle outright. For new courier riders, it was additionally possible to access a motorcycle via third-party logistics (3PL) companies linked to the e-commerce platforms—in exchange for around a quarter of their earnings on each food / grocery delivery made.^17^

Second, Samuel took advantage of almost any platform opportunity he deemed worthy, doing stints with different ride-hailing and e-commerce companies to figure out where the most lucrative spaces of boda work existed at the time—in much the same way as he had previously done as a young teacher, constantly seeking out the next best thing. Such an approach is by no means accessible to all in the sector, requiring as it does both a number of initial conditions or endowments to be in place—each platform has its own particular set of joining requirements, ranging from a good level of English to ownership of a smartphone meeting minimum standards—as well as the time and knowledge necessary to scope out all potential options.^18^ Our evidence suggests that entrance via a platform has become fairly common practice for new riders looking to start out in the sector, as borne out by both comments from longer-term riders about the nature of recent shifts as well as the personal experiences of new arrivals. One of the many consequences of the sector’s digital transition is to have expanded and in some senses simplified routes into boda work (more detail on this below), thus creating important opportunities for individuals in immediate need of new survival or supplementary income. But there is also a perception held by some that the financial realities of platform-based riding work against the city’s poorest riders, as conveyed to us by one ‘analogue’ rider of seven years: ‘They [the companies] are expensive in terms of registration, smart phone and accessories, and yet there are [also] recurring costs on airtime, data and fuel which are not accounted for in the billing systems of the digital platforms. I don’t have such money’.^19^ In this rider’s view, the majority of those joining the apps in recent years have been new to the sector and, while digitally literate, lack the traditional ‘base’ for legitimate operation—a commonly held view amongst Kampala’s longer-term riders and an issue to which we now turn.

The third insight from Samuel’s story concerns the way in which he simultaneously drew upon different forms and practices of boda work to put together a livelihood strategy that can at once be characterised as inventive yet disruptive. There have long been norms within the boda industry structuring how jobs are distributed, ensuring some degree of organisation and certainty in a largely (formally) unregulated labour market. Riders typically attach themselves to a fixed location called a ‘stage’, which is usually positioned in a site that enhances exposure to potential customers: that might be outside malls or markets, at key junctions and intersections, or within one of the city’s many local neighbourhoods. According to the sector’s most influential yet informal rules, apart from in exceptional circumstances riders should only pick up passengers and cargo from their stage. After completing the journey, they are then expected to return to the stage and wait for the next job (within the stage itself, there is a further order to how jobs are allocated). While there have always been some who operate entirely outside this system in order to maximise their personal incomes (known locally as *lubyanza*), in the process attracting a great deal of resentment and even physical abuse from riders working the ‘right’ way, with the arrival of platform work it has become increasingly straightforward and indeed normal for individuals to enter the sector as independent, stage-less riders. This was precisely the route taken by Samuel, who started out as an ‘independent contractor’ for a European food courier company before frustrations with that work led him to full-time passenger-ferrying. To access customers, he initially registered with two digital ride-hailing companies but soon found himself regularly going offline to find and negotiate directly with passengers on the street, a method he quickly realised could be used to escape the low ‘piece-rate’ payments offered by the apps. At the time of our interview with him, Samuel had three ride-hailing apps installed on his phone, which he used normally between 11am and 4pm when ‘people are very few’ and logging on can help riders tap into the diminished pool of customers, and combined this with substantial shifts of offline, stage-less work during the much busier morning and evening periods—essentially taking the most lucrative strands of work in the sector and tying them together to form a ‘mobile and recombinant strategy of survival’ (du Toit & Neves, 2014).

Samuel’s story is not universal. Not every new rider who has entered the sector as a result of COVID-related pressures earns at the same level as him. Neither have all new entrants taken the same route into the business, or operate on the same kind of working arrangement. In addition, many of Kampala’s longer-term and stage-based boda riders have experimented with the apps over the past few years, and reached their own decisions about whether and how to incorporate that technology into their daily labour practices. So, although it is possible to outline different categories of boda rider within the city, the boundaries separating them are permeable and the populations they ‘contain’ are not perfectly homogenous.

At the same time, there are several elements of Samuel’s personal experience that resonate more widely, and which reveal something about the way in which the pandemic has reshaped livelihoods and lifestyles across sector and city. Uganda’s boda industry has long worked as a survival space for people lacking access to ‘cleaner’, more secure and better paid jobs elsewhere in the economy. Yet, from its origins as a repository for (primarily) unemployed young men and school dropouts living in poverty (Howe, 2003), to its evolution towards a widespread form of work that provides livelihoods for graduates and relatively dependable side-incomes for formal sector employees, for years the composition of the workforce has been gradually diversifying—despite a continued cultural fixation with the image of the unemployed, unruly youth as the sector’s embodiment. One of the many effects of the pandemic’s economic fallout is to have sped up and deepened this compositional transformation. Contractions and suspended salaries in other industries produced a fresh wave of job seekers, and limited back-up opportunities in the wider economy compelled them to approach the motorcycle as a core means of survival. In doing so, pandemic restrictions not only further cemented the status of boda riding as a broad-based (but still heavily male-dominated) source of primary or supplementary income for Ugandans from a wide range of backgrounds—ironic in itself given how much of a battering operations and incomes across the sector took during this period—but also generated new sets of deep dependencies on the work among those who never saw themselves becoming boda riders on a full-time and potentially long-term basis. This includes both people whose prior or planned engagements with the sector only ever amounted to selective and intermittent riding as part of a broader livelihood strategy, as well as others like Samuel who always kept their distance from boda work. What is particularly interesting to note is that, in some cases at least, the events of the past two years have unexpectedly recast the way that motorcycles are being incorporated into certain people’s working lives: while those who were previously doing well might once have planned to become bosses in the *kibaluwa* system, joining a vast class of owners looking to generate a new revenue stream, they now find that the income they stand to derive from the boda business comes from riding themselves rather than renting out.

Digitisation is a part of the story here that cannot be ignored. While the precise effects of increasing external and private sector engagement in the industry is a complicated subject of ongoing study, it is clear that the expansion of new technologies of work has made it easier for new individuals to join an already swelling (if partly decongested) workforce. The implications of this are mixed and ambiguous. On the one hand, opportunities provided by the platforms have helped some people weather the impacts of lockdown, creating new routes into the survival space of the boda sector and offering alternative methods of piecing together a livelihood strategy for those already in it. On the other hand, there are signs that these technologies are both segmenting and further fragmenting labour within the sector. By opening up new ways to operate independently of the traditional stage system—but this time with the backing and sheen of corporate power, somehow creating a more professionalised iteration of the much-maligned *lubyanza* figure—app companies are helping to instil a different set of norms around how work and incomes within the sector are distributed. In bypassing pre-existing institutions of entrance and operation, the shift towards platform work risks producing a digitally connected yet largely atomised class of labour within the workforce (Graham et al., 2017), members of which are not only capable of accessing a larger share of the customer base relative to their analogue counterparts (via a fusion of online and offline means), but are also ‘freed’ from the responsibilities and expectations that traditionally come with being a boda rider.

There are pressing questions here around the place and future of the stage, which for so long has anchored work within the sector whilst acting as a crucial node of informal governance. In their recent study of temporalities and solidarity in Nairobi’s boda boda industry, Ibrahim and Bize (2018: 87) speculate that the rise of ride-hailing apps represents the ‘greatest threat’ to the future of associational life and the institution of the stage (*shimo* in kiSwahili usage), dispersing workers across the city and ‘dis-locating’ connections among riders and customers. Given how Kampala’s digital riders often remark that the ‘app is their stage’ or that ‘the stage is on the phone’, it is possible that we have begun to see a partial manifestation of this threat on the streets of the Ugandan capital. On the other hand, current and ongoing efforts by parts of the state to formally regulate the industry continue to position the stage centrally in their plans, with repeated calls for a mapping and demarcation of all ‘official’ stages in the city alongside a renewed requirement for all boda riders to register with the municipal authority through one of them (Kampala Dispatch, 2022).^20^ Combined with recent actions by several of the city’s multiple boda associations to emphasise the importance of stage membership as a pre-requisite for operating in Kampala, it seems a kind of pushback against the possibility of absolute platformisation may be under way, rendering suggestions of the stage’s disappearance both premature and far from inevitable. But ultimately, only time will tell: with the city’s largest player, SafeBoda, now morphing into Uganda’s first Gojek-like ‘super app’, and European multinational Bolt searching out new revenues in the ‘frontier’ urban markets of the country’s post-conflict northern region (Ojok, 2021), it remains to be seen just how far the sector’s digital transition will go.^21^

In any case, in contexts like this the promises of platform work must be balanced against both the concrete experiences of it alongside a consideration of how digitisation reconfigures wider systems of labour, consumption and provisioning. Ride-haling and e-commerce companies have reportedly done extremely well out of the pandemic, with 2020 hailed as a ‘watershed year for [Africa’s] digital transition’ that saw some Uganda-based start-ups experience ‘triple digit’ growth (Oxford Business Group, 2021; UNCTAD, 2020; UNDP, 2021). Through links with government ministries and UN agencies, reflecting a globalised pandemic-era shift towards more concerted experimentation with public–private partnerships in relation to service provision (Bosma et al., 2020), companies have spent the past two years promising ‘double wins’ for society: supporting livelihoods in the stifled informal economy whilst enabling the wealthier middle classes to lock down safely and comfortably. ‘Platforms for recovery’, as it were. While there has not been space to fully interrogate such claims, the analysis presented here suggests there may be far more unfolding beneath the surface than the PR messaging implies.

## Conclusion

There has long been a tendency in policy and academic thinking to essentialise informal economies. To put forward ‘blanket representations’ that strip these spaces of their nuanced, messy characters and flatten difference (Meagher, 2020). While the specific thrust of these representations has shifted over time, lurching between depictions of economic informality as variously a source of poverty, entrepreneurship, criminality and—most recently—corporate opportunity, this tendency has served to filter out important variations not just between informal economies in different places (ibid.), but as we have shown here, *within* them as well.

In the context of Kampala’s huge motorcycle taxi sector, two years of extended lockdown have both revealed pre-existing layers of internal differentiation among the workforce—something which is ‘rarely addressed in discussions of informal or precarious work’ (Ibrahim and Bize, 2018: 82; see also Rizzo, 2011)—and resulted in new configurations of urban inequality. As COVID restrictions created economic pressures that emptied the sector of some of its most precarious workers, in the process cutting many thousands off from their core source of income, so too did they drive thousands more towards the motorcycle as an alternative means of livelihood in the face of job losses in the wider economy. At its centre, this ‘substitution effect’ that has taken place over the past two years is one particular demonstration of inequality at work: those in the poorest and most vulnerable positions were forced to exit, those with greater economic stability stayed put, and those with resources and capacities entered. It bears noting of course that while many of the sector’s new entrants occupy an advantaged position relative to those who lost access to the motorcycle, they are still nowhere near the top-end of the city’s economic pecking order.

As well as revealing how a particular kind of inequality works in concrete terms, the substitution effect discussed here has also generated a new kind of unevenness in Kampala’s boda boda sector. Though it is difficult to determine the exact scale of the processes outlined in this paper, from the evidence available it is possible that we are seeing the contours of a new class of boda labour in the making. Many new entrants have been able to gain access to motorcycles with relative ease and come from elevated starting points compared to many of the sector’s longer-term and more embedded workers, for whom riding and survival have become inextricably linked over time in the face of continuously elusive pathways out. The ability to now work digitally in the capital—of course, only an option available to those with a smartphone meeting minimum standards—simplifies routes into the sector and does away with the need to form social and financial attachments to one of the city’s many stages. At the same time, the ties that new entrants still have to their places and sectors of former / alternative employment, and the aspirations that these backgrounds have given rise to, mean that many are operating within the boda sector whilst never truly self-identifying as boda riders (and in some instances actively rejecting the title).

As COVID restrictions ease more fully and opportunities in the wider economy return, some will cease to be riders and their economic relationship with the sector will come to an end. Others like Samuel, who have seen the financial gains that can be made from riding and realised how relatively straightforward it is to jump into the work on an independent basis, will continue to be intermittently engaged with the sector. As one interviewee told us, even though he was looking forward to the reopening of schools so he could get teaching again, ‘I can’t just dump it’.^22^ For him, riding will remain as a side income on weekends and holidays, forming part of a newly diversified livelihood strategy that likely would not exist had the pandemic never occurred. Then there will be those whose return to former places of work is more uncertain, drawn in by the fast money they’ve seen can be generated and reluctant to go back to working for ‘someone else [who] pays peanuts’.^23^ At the time of writing (June 2022—4 months after the full removal of pandemic restrictions), rising commodity prices and a deepening cost of living crisis have meant that many of the city’s new riders are keeping at least one foot in the boda economy as a way to get by, while others appear to have indeed jumped in on a slightly more permanent and committed basis.^24^ In the face of persistently high levels of formal waged unemployment, ongoing concerns about the quality of jobs being created in the country, and a steady replenishment of the surplus labour population as ever more young people enter the job market each year (Angurini & Naturinda, 2022), this may be a pattern that is with us for some time yet.

Whatever the lasting specifics of this social reworking—given the ongoing and unpredictable nature of these processes, it is too soon to tell whether the workforce’s new internal unevenness will develop into a durable rather than fleeting inequality (Tilly, 1998)—the COVID crisis has reaffirmed the role of Uganda’s boda boda sector as a vital space of popular economic activity, even when it is subjected to 22 months of extended lockdown. As salaries in the wider economy dried up and more formal sectors of employment wavered, many urban residents found survival in the ‘dirty work’ of passenger ferrying and cargo delivery. Through its ability to pick up the slack when loss or uncertainty occur elsewhere, boda work exhibits a degree of dependability that further challenges prior framings of informal economic activity as marginal, transient and disconnected from core economic and political structures (Young, 2019).

But although the boda economy provides livelihoods and incomes to a huge number of people, including in times of crisis, it does not work equally for everyone. Ultimately, Kampala’s boda sector is a site of layered, multidimensional inequalities, expressed not just in the uneven distribution of earnings and guarantees of work among an ever-diversifying workforce, but also in the way that particular kinds of boda labour are being incorporated into digitally mediated systems of consumption and provisioning for the city’s growing middle class. As the sector becomes increasingly central to the workings of a new configuration of urban inequality, its vitality remains—but the question of whose interests it is really serving, and how that might be changing in an era of lockdowns and ‘digital transformation’, remains an open one.

## Footnotes

1 Reflecting vernacular usage, in this article we use the terms ‘boda boda’ and its shortened form ‘boda’ interchangeably. Following a similar logic, we also use the terms rider (rather than driver) and riding (rather than driving)—though it should be pointed out that there is no clear consensus locally around which of these is the more appropriate or accepted.

2 These estimates by Evans et al. are based on the wider revenue streams generated by boda riding—e.g. maintenance and repair work by mechanics—and assume that each driver and related industry worker supports a wife and child.

3 The hospital in question is Mulago National Referral Hospital and is located in central Kampala. It receives around 15 victims from boda crashes every day (Nyangoma, 2016), and in 2010 it was calculated that treatment of injuries caused by boda crashes consumed an astonishing 62.5% of the hospital’s surgical budget (Kigera et al., 2010).

4 A notable exception is the *Lubyanza* blog run by Tom Courtright and Geofrey Ndhogezi. See https://medium.com/lubyanza

5 The city of Kampala is comprised of five divisions, 96 parishes and 1285 wards or villages. The Greater Kampala Metropolitan Area (GKMA) includes this core area but also extends to the surrounding Mpigi, Mukono and Wakiso districts.

6 Interview with owner-rider with digital experience, 6 November 2020 (male, 35). The phrase ‘what to do’ can be read as ‘anything else to do’.

7 Rider on hire-purchase arrangement with digital experience, 1 November 2020 (male, 32).

8 Rider on hire-purchase arrangement with digital experience, 2 November 2020 (male, 22).

9 Owner-rider with no digital experience, 3 November 2020 (male, 19).

10 See previous.

11 Rider on hire-purchase arrangement with no digital experience, 3 November 2020 (male, 36).

12 Owner-rider with digital experience, 7 November 2020.

13 Former SafeBoda employee, 18 January 2022. Current SafeBoda employee, 2 February 2022.

14 Rider on hire-purchase arrangement with digital experience, 8 November 2020 (male, 27).

15 Rider on hire-purchase arrangement with digital experience, 2 November 2020 (male, 39).

16 Interview on 21 January 2022. Not real name.

17 Rider on hire-purchase agreement with digital (e-commerce) experience, 16 February 2022 (male, 33).

18 Based on multiple interviews for this project, it is common for riders without any digital experience to say the reason they have never signed up to an app is simply because they do not know how the system works or what the joining process involves.

19 Owner-rider with no digital experience, 6 November 2020 (male, 26).

20 There is a long, complicated and contentious relationship between Kampala’s boda industry and formal state regulation, and these recent efforts to ‘restore sanity’ within the sector are by no means the first of their kind (Doherty, 2020; Goodfellow, 2015; Muwanga et al., 2020).

21 As well as mirroring Gojek’s super app model, SafeBoda has also previously received funding from Go-Ventures—the investment arm of the now huge Indonesian platform company—alongside more recent financial support from Google’s Africa Investment Fund.

22 Rider on hire-purchase arrangement with digital experience, 13 October 2021 (male, 34).

23 Owner-rider with digital experience, 17 November 2021 (male, 33).

24 There are certain parallels here with recent reports that some workers in the UK, including those in full-time waged employment, have started taking on additional work as couriers for gig economy platforms in their ‘free’ time (alongside other piece-rate forms of income generation) as a way of managing rising living costs (Hall, 2022).
